# Local Income Inequality, Individual Socioeconomic Status, and Unmet Healthcare Needs in Ohio, USA

**DOI:** 10.1089/heq.2017.0058

**Published:** 2018-04-01

**Authors:** Dmitry Tumin, Michelle Menegay, Emily A. Shrider, Michael Nau, Rachel Tumin

**Affiliations:** ^1^ Department of Pediatrics, The Ohio State University College of Medicine, Columbus, Ohio.; ^2^ The Ohio Colleges of Medicine Government Resource Center, Columbus, Ohio.; ^3^ Division of Epidemiology, The Ohio State University College of Public Health, Columbus, Ohio.; ^4^ Department of Sociology, The Ohio State University, Columbus, Ohio.

**Keywords:** access to care, health disparities, population health

## Abstract

**Purpose:** Income inequality has been implicated as a potential risk to population health due to lower provision of healthcare services in deeply unequal countries or communities. We tested whether county economic inequality was associated with individual self-report of unmet healthcare needs using a state health survey data set.

**Methods:** Adults residents of Ohio responding to the 2015 Ohio Medicaid Assessment Survey were included in the analysis. Ohio's 88 counties were classified into quartiles according to the Gini coefficient of income inequality. The primary outcome was a composite of self-reported unmet dental care, vision care, mental healthcare, prescription medication, or other healthcare needs within the past year. Unmet healthcare needs were compared according to county inequality quartile using weighted logistic regression.

**Results:** The analytic sample included 37,140 adults. The weighted proportion of adults with unmet healthcare needs was 28%. In multivariable logistic regression, residents of counties in the highest (odds ratio [OR]=1.13, 95% confidence interval [CI]: 1.01–1.26; *p*=0.030) and second-highest (OR=1.16, 95% CI: 1.04–1.30; *p*=0.010) quartiles of income inequality experienced more unmet healthcare needs than residents of the most equal counties.

**Conclusion:** Higher county-level income inequality was associated with individual unmet healthcare needs in a large state survey. This finding represents novel evidence for an individual-level association that may explain aggregate-level associations between community economic inequality and population health outcomes.

## Introduction

Unequal access to healthcare is an important mechanism mediating socioeconomic disparities in health status. Health advantages accrue to people with higher income, greater educational attainment, and full-time employment due to, in part, better health insurance coverage and greater access to primary and specialty medical care.^1–4^ Even among adults with private insurance coverage, many have high deductibles and out-of-pocket costs, leading families to forgo needed medical care because of cost.^5^ This may initiate a vicious spiral wherein poor health itself increases the risk of unmet healthcare needs,^6^ defined as the difference between health services necessary to deal with a particular health problem and the actual services a person receives.^7^ Estimates of the prevalence of unmet healthcare needs vary according to the study population and the specific operationalization of this concept. Before the enactment of the Patient Protection and Affordable Care Act in 2010, 10% of American adults self-reported unmet healthcare needs,^8^ whereas ∼23% had no usual source of medical care.

Contemporary research on unmet healthcare needs has focused on individual-level barriers to accessing medical care, including lack of provider availability, lack of insurance coverage, transportation issues, and high cost.^10–12^ In addition to these individual characteristics, community characteristics may also limit people's ability to access medical care.^13,14^ Particularly, community economic inequality may operate through multiple pathways to influence individual risk of unmet healthcare needs. Highly unequal communities tend to have lower levels of social capital (i.e., fewer social ties among members of the community),^15^ fewer investments in public services, and a less accessible healthcare infrastructure.^16–18^ These consequences of community economic inequality may reinforce individual-level barriers to healthcare access, particularly for socially marginalized groups. However, community economic inequality (and community economic characteristics more generally) may also limit healthcare access and adversely influence health independently of people's own socioeconomic status. Limited healthcare access in economically unequal communities is a likely explanation for correlations between economic inequality levels and worse national or community health outcomes.^19–22^

An association between economic inequality and greater unmet healthcare needs is consistent with the contribution of economic inequality to worse community health, yet, few data are available on the consequences of community economic inequality for individuals' access to care. In recent studies, greater economic inequality measured at the county level was associated with more county-level preventable admissions^20^; and greater county socioeconomic hardship was associated with worse county-level measures of access to substance abuse treatment.^23^ Furthermore, higher levels of state, county, and Census tract income inequality have been correlated with higher population rates of depression,^24^ and higher state-level income inequality has been correlated with higher mortality rates.^25^ In the United States, however, even the largest national health surveys lack sufficient sample sizes at the county level to directly estimate county-specific access to healthcare.^26,27^ To assess the relationship between greater county-level economic inequality and individual unmet healthcare needs, we used a unique large state-level survey data set designed for county-level analysis. Our primary hypothesis was that adults residing in counties with greater levels of economic inequality would be more likely to report unmet healthcare needs. Our secondary aim was to assess the importance of economic inequality while accounting for individual socioeconomic characteristics that could predict unmet healthcare needs.

## Methods

Data for the study were obtained from the 2015 Ohio Medicaid Assessment Survey (OMAS), a cellphone and landline telephone survey of noninstitutionalized adults living in Ohio.^28^ Adults aged 19 years and older were invited to participate in the survey if they lived in Ohio for at least 1 month. Interviews were collected from January to June 2015. Cases with complete data on unmet healthcare needs (described further hereunder) and individual-level covariates in the study were selected for analysis. This study was exempt from Institutional Review Board approval as it was not considered human subjects research, because it involved secondary analysis of a de-identified public-use file.^29^ Data analysis included survey weights to adjust for unequal probability of sample selection, and standard errors were adjusted for the complex design of the survey sample.^28^

The outcome was a composite measure of unmet healthcare needs over a 1-year period, constructed from the following survey questions^30^:

During the past 12 months…

1. Was there a time when you needed dental care but could not get it at that time?
2. Have you not filled a prescription because of the cost? This includes refills.
3. Was there a time when you needed vision care or eye glasses, but could not get it at that time?
4. Was there a time when you needed mental healthcare or counseling services, but could not get it at that time?
5. Was there any time when you needed any other healthcare, such as a medical examination or medical supplies, but could not get it at that time?

Responses to each question were recorded as “Yes” or “No.” “Don't know” and “Refused” responses were treated as missing. To construct the composite measure of unmet healthcare needs, a score of 1 was assigned if a respondent had at least 1 type of unmet healthcare need, and a score of 0 was assigned if a respondent reported no unmet healthcare needs. Cases in which all responses to the unmet healthcare needs questions were missing were excluded from the analysis. The primary independent variable was a measure of county-level income inequality. The Gini coefficient of household incomes (ranging from 0, perfect equality, to 1, perfect inequality)^20^ was obtained for each Ohio county from the 2010 to 2014 American Community Survey.^31^ The 88 Ohio counties were then classified into quartiles according to the Gini coefficient.

Weighted logistic regression was used to evaluate the association between county inequality and individual unmet healthcare needs. The model included the measure of county inequality (Gini coefficient quartile), as well as the following individual-level covariates: age, gender, race/ethnicity (non-Hispanic white, non-Hispanic black, Hispanic, and other), educational attainment (high school or less, some college, and 4-year college degree), marital status (never married, married, separated/divorced/widowed, and unmarried couple), number of children ≤18 years in the family, current work status, insurance type (commercial [employer-sponsored or privately purchased], Medicaid, Medicare [without Medicaid], other, and none), family income as percent of the federal poverty level (FPL), self-rated health (1–5 scale from excellent to poor), cardiovascular chronic conditions (diagnosed hypertension, heart attack, coronary heart disease, or congestive heart failure), diabetes (not solely due to pregnancy), cancer, and smoking status (never smoked, former smoker, and current smoker). Income data were completed by OMAS staff using single imputation due to a high fraction of missing information.^28^ Cases with missing data on covariates were excluded from the analysis. Analyses included survey weights to account for unequal probability of sample selection, and variance estimation was adjusted for the complex survey design.^28^ All data analysis was performed using Stata/MP 13.1 (College Station, TX: StataCorp, LP), and two-sided *p*<0.05 was considered statistically significant.

## Results

Of 42,876 adult respondents to the 2015 OMAS, we excluded 2362 with missing data on unmet healthcare needs and 3374 with missing data on covariates, leaving a final sample of 37,140 respondents for descriptive and multivariable analysis. Estimated population characteristics based on this sample are summarized in Table 1. There were 10,438 (weighted proportion: 0.28; 95% confidence interval [CI]: 0.28–0.29) adults with at least one type of unmet healthcare need, and the most common types of unmet healthcare needs were related to prescription drug access (5499; weighted proportion: 0.15; 95% CI: 0.15–0.16) and dental care (4498; weighted proportion: 0.12, 95% CI: 0.12–0.13). Respondents in the survey were primarily non-Hispanic white (84%). Half were currently married, while a majority (56%) had completed at least some college education. Health insurance coverage was predominantly private (51%), followed by Medicare (20%), Medicaid (18%), and other types of coverage (4%). Seven percent had no current health insurance coverage, while 16% had household incomes <100% FPL.

**Table 1.** **Weighted Proportions or Means of Study Variables Among Adult Respondents to the 2015 Ohio Medicaid Assessment Survey (*N*=37,140)**

Variable	Proportion or mean	95% CI^a^
Unmet healthcare needs		
Dental care	0.12	(0.12–0.13)
Prescription medications	0.15	(0.15–0.16)
Vision care	0.11	(0.10–0.11)
Mental health	0.04	(0.04–0.04)
Other	0.07	(0.07–0.08)
Any unmet healthcare needs^b^	0.28	(0.28–0.29)
Age (years)	48.4	(48.1–48.6)
Female	0.52	(0.51–0.52)
Race/ethnicity		
Non-Hispanic white	0.84	(0.83–0.84)
Non-Hispanic black	0.12	(0.11–0.12)
Hispanic	0.03	(0.02–0.03)
Other	0.02	(0.02–0.03)
Educational attainment		
High school or less	0.44	(0.43–0.45)
Some college or 2-year degree	0.31	(0.30–0.32)
Four-year college degree	0.25	(0.24–0.25)
Marital status		
Never married	0.23	(0.22–0.23)
Married	0.50	(0.49–0.51)
Separated/divorced/widowed	0.23	(0.22–0.23)
Unmarried couple	0.05	(0.04–0.05)
Children in family		
0	0.65	(0.65–0.66)
1	0.14	(0.14–0.14)
2	0.12	(0.12–0.13)
3 or more	0.08	(0.08–0.09)
Currently working	0.60	(0.59–0.61)
Health insurance coverage		
Private	0.51	(0.50–0.52)
Medicaid	0.18	(0.18–0.19)
Medicare^c^	0.20	(0.20–0.21)
None	0.07	(0.06–0.07)
Other	0.04	(0.04–0.04)
Household income (% FPL)		
<100%	0.16	(0.16–0.17)
100 to <200%	0.22	(0.22–0.23)
200 to <300%	0.18	(0.17–0.18)
300 to <400%	0.14	(0.13–0.14)
400% or greater	0.30	(0.29–0.30)
Self-rated general health		
Excellent	0.19	(0.18–0.19)
Very good	0.34	(0.33–0.35)
Good	0.29	(0.33–0.35)
Fair	0.14	(0.13–0.14)
Poor	0.04	(0.04–0.05)
History of cardiovascular chronic disease^d^	0.37	(0.37–0.38)
History of diabetes	0.13	(0.13–0.14)
History of cancer	0.11	(0.10–0.11)
Smoking status		
Never smoked	0.53	(0.53–0.54)
Former smoker	0.24	(0.24–0.25)
Current smoker	0.23	(0.22–0.23)

^a^ Model variance adjusted for complex sampling design.

^b^ Primary outcome variable in multivariable logistic regression analysis.

^c^ Not including respondents who also have Medicaid coverage.

^d^ Ever diagnosed with hypertension, heart attack, coronary heart disease, or congestive heart failure.

CI, confidence interval; FPL, federal poverty level.

**FIG. 1.**
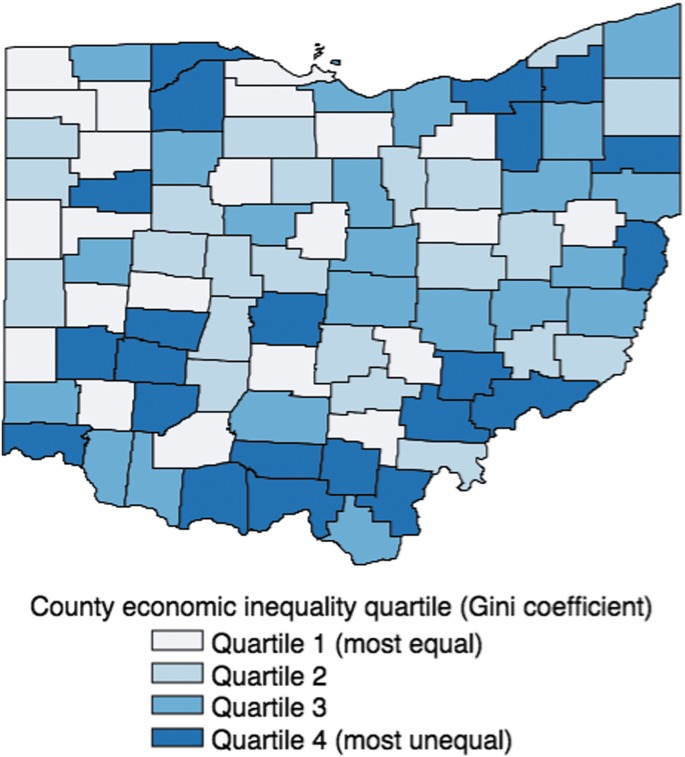
Ohio counties classified by quartile of income inequality (Gini coefficient).

**FIG. 2.**
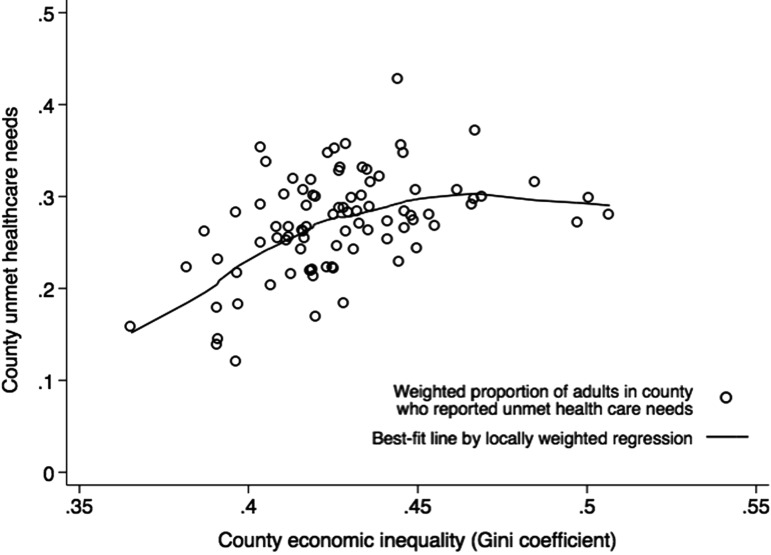
Joint distribution of county-level income inequality (Gini coefficient) and unmet healthcare needs, with best-fit line determined by locally weighted regression.

All 88 Ohio counties were represented in the analytic sample, and county-level sample sizes ranged from 56 to 3740 respondents. County-level Gini coefficients ranged from 0.37 to 0.51, and county quartiles of income inequality are illustrated in Figure 1. County weighted proportions of adults with unmet healthcare needs are plotted against the county-level Gini coefficient in Figure 2. These aggregate data show that unmet healthcare needs increase linearly with county level of economic inequality (Pearson's *r*=0.45), excepting three outlier counties with the highest levels of inequality, but moderately high proportions of unmet healthcare needs (Athens, Cuyahoga, Hamilton). Two of these outlier counties are major urban centers: Cleveland (Cuyahoga) and Cincinnati (Hamilton).

The individual-level logistic regression model is shown in Table 2. Compared to residents of counties in the lowest quartile of income inequality (most equal), residents of counties in the second-highest and highest quartiles had greater odds of reporting unmet healthcare needs, after adjustment for individual demographic, economic, and health characteristics (odds ratio [OR]=1.16; 95% CI: 1.04–1.30; *p*=0.010 for the second-highest quartile; and OR=1.13; 95% CI: 1.01–1.26; *p*=0.030 for the highest quartile). Among individual-level covariates in the analysis, black race, lower household income, lack of health insurance, and being unmarried were associated with higher odds of unmet healthcare needs. Worse respondent-rated health, previous diagnosis of chronic disease, and a history of smoking were also associated with greater odds of unmet healthcare needs.

**Table 2.** **Weighted Multivariable Logistic Regression Model of Unmet Healthcare Needs Among Adult Respondents to the 2015 Ohio Medicaid Assessment Survey (*N*=37,140)**

Variable	OR	95% CI^a^	*p*^a^
County income inequality quartile^b^			
1 (most equal)	Ref.		
2	1.03	(0.91–1.17)	0.621
3	1.16	(1.04–1.30)	0.010
4 (most unequal)	1.13	(1.01–1.26)	0.030
Age (years)	0.98	(0.98–0.98)	<0.001
Female	1.44	(1.35–1.54)	<0.001
Race/ethnicity			
Non-Hispanic white	Ref.		
Non-Hispanic black	1.23	(1.12–1.36)	<0.001
Hispanic	0.88	(0.72–1.08)	0.224
Other	1.16	(0.91–1.48)	0.230
Educational attainment			
High school or less	Ref.		
Some college or 2-year degree	1.32	(1.23–1.42)	<0.001
Four-year college degree	1.26	(1.16–1.38)	<0.001
Marital status			
Never married	Ref.		
Married	1.23	(1.11–1.35)	<0.001
Separated/divorced/widowed	1.39	(1.25–1.55)	<0.001
Unmarried couple	1.37	(1.16–1.61)	<0.001
Children in family			
0	Ref.		
1	1.01	(0.91–1.12)	0.825
2	1.00	(0.89–1.12)	0.984
3 or more	0.87	(0.76–0.99)	0.038
Currently working	0.93	(0.86–1.01)	0.076
Health insurance coverage			
Private	Ref.		
Medicaid	0.87	(0.79–0.97)	0.013
Medicare^c^	0.92	(0.83–1.02)	0.129
None	2.47	(2.17–2.82)	<0.001
Other	1.03	(0.86–1.24)	0.756
Household income (% FPL)			
<100%	Ref.		
100 to <200%	1.00	(0.91–1.11)	0.935
200 to <300%	0.73	(0.65–0.82)	<0.001
300 to <400%	0.59	(0.52–0.68)	<0.001
400% or greater	0.47	(0.42–0.53)	<0.001
Self-rated general health			
Excellent	Ref.		
Very good	1.61	(1.45–1.80)	<0.001
Good	2.58	(2.31–2.89)	<0.001
Fair	4.24	(3.73–4.83)	<0.001
Poor	6.62	(5.58–7.87)	<0.001
History of cardiovascular chronic disease^d^	1.16	(1.07–1.25)	<0.001
History of diabetes	1.16	(1.06–1.26)	0.002
History of cancer	1.08	(0.98–1.20)	0.119
Smoking status			
Never smoked	Ref.		
Former smoker	1.25	(1.16–1.35)	<0.001
Current smoker	1.45	(1.34–1.58)	<0.001

^a^ Model variance adjusted for complex sampling design.

^b^ Calculated as Gini coefficient from 2010 to 2014 American Community Survey data.

^c^ Not including respondents who also have Medicaid coverage.

^d^ Ever diagnosed with hypertension, heart attack, coronary heart disease, or congestive heart failure.

OR, odds ratio.

## Discussion

Greater economic inequality is associated with worse health through multiple mechanisms, including increased social distances between rich and poor people residing in the same community, and increased disparities in access to healthcare.^16,17,32^ Previous analyses of aggregate data found that county-level measures of access to care were worse in counties with higher economic inequality. However, individual-level evidence on the effects of local economic inequality is scarce. Using a state health survey uniquely designed for inference at the county level, the present study demonstrates that unmet healthcare needs are more common among residents of counties with higher levels of economic inequality than among residents of economically equal counties. Despite strong associations between our study outcome and individual income, educational attainment, and availability of health insurance coverage, county economic inequality emerged as a contextual characteristic independently associated with the risk of unmet healthcare needs. This finding supports the importance of addressing economic inequality as part of a strategy for improving access to healthcare.

Income inequality in the United States has grown rapidly over the last 30 years, reaching its highest levels since the early 1900s.^21,33^ At the national level, greater economic inequality correlates with diminished life expectancy.^34^ Within the United States, individual exposure to unequal communities is associated with worse health among adolescents^19^; while counties with greater economic inequality have higher rates of depression and poor self-rated health.^20^ Access to care is considered to mediate the health consequences of economic inequality, such that greater inequality reduces individual access to care, and individual unmet healthcare needs increase the risk of morbidity, mortality, and poor health.^35,36^ Consistent with this reasoning, the present study confirms that unmet healthcare needs are greater among adults residing in less-equal counties, even adjusting for individual socioeconomic characteristics that influence access to care, and individual health characteristics that influence demand for primary and specialty care. Interestingly, the association of county inequality with individual unmet healthcare needs was not linear, with respondents in the top 2 most unequal quartiles having similar levels of unmet healthcare needs. In the top quartile, county income inequality may be confounded with urbanization and the presence of major academic hospital systems. As shown in Figure 1, counties in the top quartile of income inequality include a heterogeneous mix of Ohio's major cities as well as Appalachian counties in the state's Southeast. Therefore, further work is needed to clarify when extreme economic inequality may especially limit individuals' access to healthcare. Together with the present results on inequality and access to care, this research would clarify the mechanisms underlying patterns seen in aggregate county-, year-, or nation-level data.^37^

In addition to county economic inequality, the present analysis identified several individual-level factors associated with greater risk of unmet healthcare needs. Some of these factors clearly relate to a greater need for healthcare services—specifically, worse self-rated health, history of chronic disease, and history of smoking. Other factors associated with greater risk of unmet healthcare needs represented multiple measures of socioeconomic disadvantage. Particularly, we confirmed greater risk of unmet healthcare needs among black compared with white respondents; among respondents without health insurance coverage compared with respondents with public or private health insurance; and among respondents with lower household incomes. These findings are consistent with previous research, although specific causal pathways linking these socioeconomic characteristics to unmet healthcare needs could not be discerned in the present cross-sectional study.^10–12,38^ A novel and unexpected result was the increased likelihood of reporting unmet healthcare needs among people with higher educational attainment. While higher educational attainment is generally considered a signifier of socioeconomic advantage and predicts improved access to care,^6^ more educated people may also be more likely to seek or demand medical care due to higher health literacy, creating more potential for unmet healthcare needs.

An important strength of the present analysis is the use of a unique health survey data set. The 2015 OMAS originally included over 40,000 adult respondents, with at least 56 respondents per county in the final analytic sample. By comparison, the 2015 Behavioral Risk Factor Surveillance System—the largest health survey fielded in the United States—included <12,000 respondents from Ohio.^39^ The sample size of the 2015 OMAS supported estimating the association between county characteristics and individual outcomes,^38^ in contrast to previous research that focused on county-level outcomes of healthcare utilization.^20^ However, the design of the OMAS introduced some limitations to this analysis. First, the survey was cross-sectional, precluding causal inference about the accumulation of exposure to county-level economic inequality.^19^ In addition, we cannot definitively identify which respondents have lived in their current county for the entire 1-year period over which unmet healthcare needs were assessed, resulting in potential bias when attributing individual unmet healthcare needs to county characteristics. However, the extent of this potential bias is limited by the low annual rate (<4%) of moves between counties in the United States.^40^

Other limitations of our study include analysis of OMAS data using single-level logistic regression, to incorporate the recommendation that variances be adjusted for the complex sampling design.^28^ This adjustment took into account nonindependence of outcomes among members of the same stratum; but strata definitions did not match county boundaries,^28^ meaning that the residual variability in unmet healthcare needs due to unobserved county-level characteristics could not be estimated. Furthermore, the Gini coefficient was used as a summary measure of inequality, for consistency with recent research on county-level aggregate healthcare utilization^20^; future research should consider alternative measures of county-level socioeconomic characteristics as potential correlates of greater individual unmet healthcare needs.^38,41^ Finally, our analysis adjusted for self-reported measures of health status and health behavior, such as self-reported general health, but could not account for potential bias or error in respondents' reporting of their own health.^42^

## Conclusion

Income inequality has been implicated in perpetuating health disparities through its effects on the provision of healthcare. While previous studies have demonstrated correlations between higher local income inequality and aggregate indicators of diminished access to care, the lack of sufficient data within small geographic units prevented confirmation of the underlying individual-level effect. Using a unique state health survey data set, the present study offers the first evidence that greater county income inequality is associated with individual adults' higher risk of unmet healthcare needs, after adjustment for a comprehensive range of individual-level confounding variables. Furthermore, unmet healthcare needs were more common among black respondents, unmarried respondents, respondents without health insurance coverage, and respondents with lower household income. These findings provide empirical foundation for policies aiming to improve equitable access to care by targeting healthcare provision in communities with polarized income or wealth distributions.

## Abbreviations Used

CI: confidence interval
FPL: federal poverty level
OMAS: Ohio Medicaid Assessment Survey
OR: odds ratio
